# Association of a Triage-Based Emergency Alarm System with Waiting Times and Clinical Outcomes in a Pediatric Emergency Department

**DOI:** 10.3390/children13101331

**Published:** 2026-09-30

**Authors:** Yi-Jung Chang, Mei-Chuan Shih, Chien-Chung Lee

**Affiliations:** 1 Department of Pediatrics, Chang Gung Memorial Hospital, College of Medicine, Chang Gung University, Taoyuan 33373, Taiwan; 2 Department of Nursing, Linkou Chang Gung Memorial Hospital, Taoyuan 33373, Taiwan

**Keywords:** pediatric emergency department, triage, emergency alarm, waiting time, crowding, COVID-19, patient outcomes

## Abstract

**Highlights:**

**What are the main findings?**

- A triage-triggered emergency alarm was associated with a shorter median waiting time for triage level 1 patients.
- No statistically significant difference in triage level 2 waiting time was detected in the matched-pair analysis.

**What are the implications of the main findings?**

- A simple automated visual alarm may help pediatric emergency departments prioritize critically ill children during periods of crowding.
- The intervention may be associated with earlier electronic documentation of initial physician evaluation without substantially delaying care for other emergent patients.

**Abstract:**

**Background/Objectives**: Pediatric emergency departments (PEDs) experience abrupt fluctuations in patient volume that can prolong waiting times and compromise timely care for critically ill children. The aim of this study was to evaluate whether implementation of a triage-based emergency alarm was associated with time to electronic documentation of initial physician evaluation and clinical outcomes during the 2022 COVID-19 Omicron wave in Taiwan. **Methods**: We conducted a single-center retrospective before-and-after observational study of PED visits from 1 April to 31 October 2022. The emergency flashing-light alarm was implemented on 1 August 2022 to alert physicians to triage level 1 patients requiring immediate evaluation. Propensity scores were estimated using age group (<5 versus 5–17 years), sex, and availability of additional physician staffing support; 1:1 matching yielded 7467 pairs. The matched-pair structure was retained in bootstrap confidence intervals and permutation tests for waiting-time comparisons. **Results**: The analysis included 19,624 visits (10,814 before and 8810 after implementation). In the matched cohort, median waiting time among triage level 1 visits was 11.0 min (IQR, 6.8–17.2) before and 8.2 min (IQR, 5.7–12.5) after implementation; the post-minus-pre difference between subgroup medians was −2.82 min (95% CI, −3.48 to −1.80; *p* < 0.001). Among triage level 2 visits, medians were 12.4 and 11.8 min, respectively (*p* = 0.088). Hospital admission and PICU admission or planned admission were more frequent after implementation. **Conclusions**: The alarm was associated with a shorter median time to documented initial physician evaluation among triage level 1 visits. No statistically significant difference was detected for triage level 2 waiting time in the matched-pair analysis. Residual differences in triage mix and secular trends preclude causal inference.

## 1. Introduction

Emergency department (ED) crowding is a worldwide healthcare problem and an important patient safety concern that intensified during the COVID-19 pandemic [1]. Surges in patient volume, constrained resources, and staffing shortages may amplify the adverse effects of crowding [2]. ED crowding has been associated with delayed care, reduced quality, medical errors, higher costs, and lower patient and clinician satisfaction [1,3]. During the pandemic, hospital strain was also associated with excess mortality [4]. Although the World Health Organization ended the ‘public health emergency of international concern’ status of COVID-19 in May 2023, regional surges in pediatric respiratory illness have continued to challenge pediatric emergency departments (PEDs) [5,6].

Pediatric demand is particularly variable. Influenza seasons and outbreaks of contagious diseases can rapidly increase PED attendance, while physiologic vulnerability and seasonal environmental exposures may precipitate respiratory and other acute illnesses [7,8]. At the same time, many PED visits involve noncritical conditions, reflecting parental concern, uncertainty about illness severity, and limited access to primary care. Effective triage is therefore essential to identify children who require immediate evaluation and to allocate limited resources according to clinical urgency.

The COVID-19 pandemic disrupted ED operations worldwide despite established triage and crowding protocols [9,10,11]. Existing flow interventions have often focused on low-acuity diversion, fast-track pathways, staffing expansion, or broader triage redesign. Evidence regarding simple, point-of-triage operational interventions that direct clinicians immediately to the most critically ill children remains limited. In our PED, triage classification alone was entered into the information system and did not provide a conspicuous real-time signal in the physician work area. On 1 August 2022, the PED therefore introduced an automated flashing-light alarm triggered by the onset of triage level 1. The alarm was intended to convert the triage designation into an immediate visual notification and reduce the interval between completed triage and physician assessment. The aim of this study was to evaluate whether implementation of this triage-triggered emergency alarm was associated with time to electronic documentation of initial physician evaluation among triage level 1 patients and with selected clinical and balancing outcomes.

## 2. Materials and Methods

### 2.1. Study Design and Setting

We conducted a retrospective, single-center, before-and-after observational study in the pediatric emergency department of Chang Gung Memorial Hospital, Linkou Branch, Taoyuan, Taiwan, a level I tertiary care academic hospital located in northern Taiwan, with an annual PED census of approximately 40,000 visits during the study period. The study period extended from 1 April to 31 October 2022 and encompassed changing phases of the 2022 SARS-CoV-2 Omicron wave in Taiwan. The pre-intervention period was 1 April–31 July 2022, and the post-intervention period was 1 August–31 October 2022. Because these periods occurred at different points in the epidemic trajectory and in different calendar months, secular and seasonal effects were considered important potential sources of residual confounding. Monthly PED visit volume and triage-level distribution across the full study period are presented descriptively in Supplementary Table S1.

### 2.2. Participants

**Figure 1:**
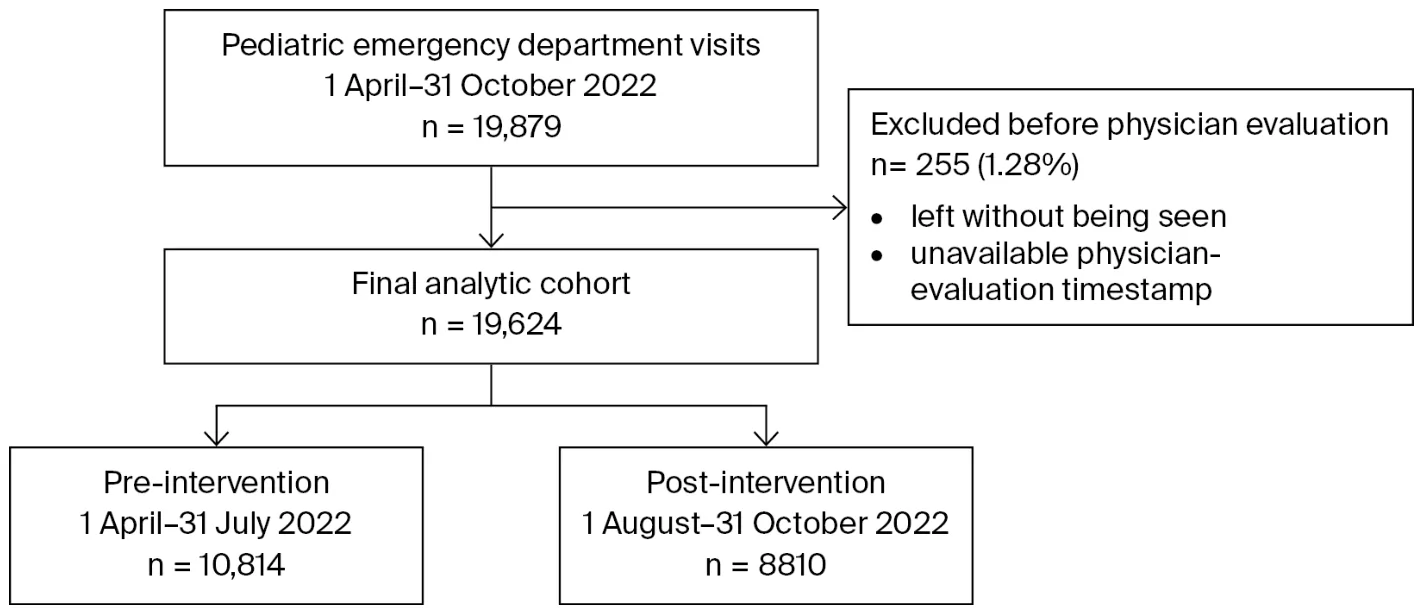
Study participant selection and analysis flow.

During the study period, 19,879 pediatric emergency department visits were recorded. Visits were eligible if a physician-evaluation timestamp was available for calculation of the primary waiting-time outcome. A total of 255 visits (1.28%) were excluded because the patients left before physician evaluation (left without being seen), resulting in an unavailable physician-evaluation timestamp. The final analytic cohort therefore comprised 19,624 visits. A complete-case approach was used for the primary outcome. Among included visits, no additional missing values were identified for the variables used in the analyses, and no imputation was performed. The unit of analysis was the PED visit; therefore, the same child could contribute more than one visit during the study period. Repeated visits were not linked at the patient level, and potential within-patient correlation was not modeled. The participant-selection process is shown in Figure 1.

### 2.3. Triage Classification and Intervention

Patients were classified using the pediatric criteria of the Taiwan Triage and Acuity Scale (TTAS). Triage level 1 (resuscitation) comprised immediately life-threatening conditions requiring immediate physician assessment and resuscitation. Representative criteria included severe respiratory distress or respiratory failure, airway obstruction, oxygen saturation below 90%, shock or severe perfusion failure, capillary refill time longer than 4 s, unconsciousness or a Glasgow Coma Scale score of 3–8, inability to protect the airway, ongoing seizures, and a core temperature above 41 °C or below 32 °C. Triage level 2 (emergent) comprised potentially unstable conditions requiring physician assessment within 10 min. Representative criteria included moderate respiratory distress with increased work for breathing or accessory-muscle use, oxygen saturation below 92%, marginal hemodynamic perfusion without overt shock, capillary refill time longer than 2 s, acute altered mental status or a Glasgow Coma Scale score of 9–13, a recently terminated seizure, marked weakness, and age-specific temperature abnormalities or ill appearance. The final level was assigned using the overall clinical assessment rather than any single physiologic threshold. The TTAS definitions and recommended response targets remained unchanged throughout the study.

Beginning on 1 August 2022, the emergency flashing-light alarm was integrated into the electronic triage workflow. The triage nurse assessed the patient and finalized the triage level in the hospital information system. Entry of a triage level 1 patient automatically activated the alarm; no separate manual activation was required. A red warning light mounted on the wall of the physician consultation area flashed to notify the physicians on duty. Opening the patient chart automatically deactivated the light, without a manual override. Chart opening and documentation of the initial physician evaluation were distinct system events. The timestamp used for the primary outcome was the electronic documentation time of the initial physician evaluation, not the chart-opening time that deactivated the alarm. The established triage level 2 workflow and 10 min assessment target were unchanged.

Before implementation, triage nurses and physicians were informed of the new alarm workflow through a departmental announcement. No additional formal triage training was conducted. Staff were informed that a flashing red light indicated a triage level 1 patient requiring immediate physician assessment and electronic documentation. Alarm activation and deactivation occurred automatically within the information system; however, the event-log variables required to verify activation for every eligible visit and calculate the interval from alarm activation to chart access were not extracted into the research dataset for the full cohort. Consequently, implementation fidelity and activation-to-chart-access response time were not assessed.

### 2.4. Data Sources and Outcomes

Data were extracted from the hospital electronic health record and hospital computer logs. Variables included age, sex, visit date and time, triage level, additional physician staffing support, time from triage to initial physician evaluation, hospital admission, PICU admission or planned admission, and death occurring in the PED. Additional physician staffing support was defined as the availability of an additional physician assigned to support PED clinical care during the patient’s visit. PICU admission or planned admission was defined as either completed PICU admission or a documented decision for PICU admission when transfer could not be completed because the patient experienced in-hospital cardiac arrest (IHCA) in the PED. This composite was used to retain critically ill patients for whom PICU transfer was intended but could not be completed. Because planned admission may be related to the mortality outcome itself, mortality analyses using this denominator were considered exploratory and susceptible to selection bias. Response time was defined as the interval from final entry of the triage level by the triage nurse to electronic documentation of the initial physician evaluation. This documentation timestamp was system-recorded and distinct from the chart-opening event that deactivated the alarm. However, it may not correspond exactly to the beginning of bedside assessment, and no independent bedside timestamp was available consistently for the full cohort.

The primary outcome was time from triage to electronic documentation of initial physician evaluation among triage level 1 patients. Secondary outcomes included triage level 2 waiting time, hospital admission, PICU admission or planned admission, and PED mortality among patients with actual or planned PICU admission. The mortality analysis was exploratory.

### 2.5. Statistical Analysis

Statistical analyses were performed using IBM SPSS Statistics, version 24.0 (IBM Corp., Armonk, NY, USA), with additional paired analyses of the matched dataset. Waiting times are presented as medians and interquartile ranges (IQRs) because their distributions were right-skewed. The propensity score was estimated from three binary variables: age group (<5 versus 5–17 years), sex, and availability of additional physician staffing support. Pre- and post-intervention visits were matched 1:1 on the propensity score without reuse of visits; the retained pairs had the same propensity score and the same values of all three model variables. The extracted dataset contained reciprocal visit identifiers that allowed all 7467 pairs to be reconstructed uniquely. Covariate balance was assessed with standardized mean differences (SMDs), calculated as the post-minus-pre difference in means or proportions divided by the pooled standard deviation; an absolute SMD < 0.1 was used as a descriptive balance threshold. Arrival times were divided into 00:00–14:59 and 15:00–23:59 because the latter nine-hour period had a higher concentration of PED arrivals per hour. This operational grouping was used to describe the arrival-time distribution and was not included in the propensity score model. Triage level was also not included in the propensity score model. For waiting-time comparisons in the matched cohort, the effect measure was the difference between the post- and pre-intervention subgroup medians. Its percentile 95% confidence interval was estimated by resampling entire matched pairs 10,000 times, recalculating the subgroup medians in each replicate. Two-sided *p*-values were obtained from 20,000 within-pair label permutations. Because both visits in a pair were not necessarily assigned the same triage level, these are differences between the triage-specific marginal medians in the matched cohort, not medians of within-pair waiting-time differences. Matched binary hospitalization and actual or planned PICU admission outcomes were compared using exact McNemar tests. Unmatched comparisons used the Mann–Whitney U test for waiting times and the chi-square or Fisher’s exact test for categorical outcomes, as appropriate. The rare-event PED mortality comparison among visits with actual or planned PICU admission was exploratory; its Fisher exact analysis does not fully account for matching, and it was not interpreted as a confirmatory treatment effect. No interrupted-time-series analysis was performed. Missing data were handled by complete-case analysis, without imputation. All tests were two-sided, with *p* < 0.05 considered statistically significant. As a sensitivity analysis addressing outcome-dependent selection, PED mortality was summarized after restricting the matched subgroup to patients with completed PICU admission.

### 2.6. Ethical Approval

The study was conducted in accordance with the Declaration of Helsinki and approved by the Institutional Review Board of Chang Gung Memorial Hospital (protocol code 202201048B0). The requirement for individual informed consent was waived because of the retrospective design and use of de-identified data. This retrospective observational study used existing medical records. The study period began on 1 April 2022, which indicates the date from which routinely collected clinical data were retrospectively reviewed and does not represent the initiation of participant enrollment or prospective research procedures. No participants were prospectively recruited, and no research-specific intervention was conducted before ethics approval was obtained.

## 3. Results

### 3.1. Study Population

From 1 April to 31 October 2022, the PED recorded 19,879 visits. Of these, 255 visits (1.28%) were excluded because the patients left before physician evaluation and therefore there was no physician-evaluation timestamp. The exclusion proportion was similar in the pre-intervention period (139/10,953, 1.27%) and post-intervention period (116/8926, 1.30%). These excluded visits had no physician evaluation or subsequent disposition; therefore, admission, PICU admission, and PED mortality outcomes could not be compared with those of included visits. Detailed individual-level characteristics of excluded visits were not retained in the analytic dataset, precluding assessment of whether the missingness mechanism was random. The final analytic cohort included 19,624 visits: 10,814 in the pre-intervention period and 8810 in the post-intervention period. After 1:1 propensity score matching, 7467 visits remained in each group (Figure 1); 3347 pre-intervention visits and 1343 post-intervention visits were unmatched. Most children were younger than 5 years. A slight majority of visits occurred from 00:00 to 14:59, rather than from 15:00 to 23:59. The arrival-time groups reflect the higher hourly concentration of visits during 15:00–23:59; Table 1 counts were recalculated from recorded arrival timestamps. Before matching, children in the pre-intervention period were older, additional physician staffing support was more frequently available before implementation, and triage-level distributions differed between periods and remained different after matching. The SMDs for the three matched binary covariates were 0 after matching, whereas those for triage levels 1 and 3 were −0.125 and 0.196, respectively. Characteristics before and after matching are summarized in Table 1.

### 3.2. Waiting Times and Clinical Outcomes

Among matched triage level 1 visits (883 before and 605 after implementation), median waiting time decreased from 11.0 min (IQR, 6.8–17.2) to 8.2 min (IQR, 5.7–12.5). The post-minus-pre difference between subgroup medians was −2.82 min (pair-bootstrap 95% CI, −3.48 to −1.80; paired permutation *p* < 0.001). Only 79 matched pairs had triage level 1 visits in both periods; the reported effect therefore compares the marginal medians for all triage level 1 visits retained after matching, rather than a within-pair median difference. Among matched triage level 2 visits, the corresponding medians were 12.4 min (IQR, 7.3–21.5) and 11.8 min (IQR, 7.2–19.9); the difference was −0.58 min (pair-bootstrap 95% CI, −1.37 to 0.15; paired permutation *p* = 0.088). Hospital admission (964 versus 1210; exact McNemar *p* < 0.001) and PICU admission or planned admission (87 versus 139; exact McNemar *p* < 0.001) were more frequent after implementation in the matched cohort (Table 2). These utilization outcomes may reflect case mix or practice changes and do not by themselves establish increased clinical severity.

Before matching, PED mortality among patients with actual or planned PICU admission was 7/115 (6.1%) in the pre-intervention period and 2/153 (1.3%) in the post-intervention period (*p* = 0.040). After matching, the corresponding values were 5/87 (5.7%) and 2/139 (1.4%), respectively. For the post- versus pre-intervention comparison, the exact conditional odds ratio was 0.24 (95% CI, 0.02–1.51; *p* = 0.110), and the absolute risk difference was −4.3 percentage points (95% CI, −11.4 to 0.6 percentage points). In the matched cohort, five patients before implementation and two patients after implementation were classified as planned PICU admissions because they experienced IHCA in the PED before transfer could be completed; all seven died in the PED. After excluding these planned admission/IHCA cases, 82 pre-intervention patients and 137 post-intervention patients had completed PICU admissions. In this sensitivity analysis, PED mortality was 0/82 (0%) before and 0/137 (0%) after implementation, precluding estimation of a comparative effect. Matching excluded 3347 pre-intervention and 1343 post-intervention visits, including two pre-intervention deaths and no post-intervention deaths. Among unmatched patients with actual or planned PICU admission, PED mortality was 2/28 (7.1%) before implementation and 0/14 (0%) after implementation. Compared with matched visits, unmatched visits had lower hospitalization rates before implementation (382/3347 [11.4%] vs. 964/7467 [12.9%]) and after implementation (204/1343 [15.2%] vs. 1210/7467 [16.2%]). Actual or planned PICU admission was also less frequent among unmatched visits before implementation (28/3347 [0.8%] vs. 87/7467 [1.2%]) and after implementation (14/1343 [1.0%] vs. 139/7467 [1.9%]). These utilization indicators suggest somewhat lower overall acuity among unmatched visits, although detailed physiologic and diagnostic data were unavailable to establish systematic differences in clinical severity. The removal of two pre-intervention deaths, together with the smaller number of events and reduced precision, explains the divergence between the unmatched and matched analyses. These mortality results were not restricted to triage level 1 visits and did not include deaths after transfer from the PED.

## 4. Discussion

In this single-center before-and-after study, implementation of a triage-based emergency alarm was associated with a shorter time to documented initial physician evaluation among triage level 1 visits. The difference between the matched-cohort subgroup medians was −2.82 min (95% CI, −3.48 to −1.80). For triage level 2 visits, the estimated difference was small and its paired analysis did not reach statistical significance. These findings suggest a shorter interval to documented evaluation for the most urgent group without evidence of a substantial delay for the next-highest urgency group. However, triage level was not included in the propensity score model, and differences in triage mix persisted after matching. No independent bedside timestamp was available, so the association may partly reflect earlier electronic documentation rather than earlier bedside assessment. Supplementary Table S1 describes monthly changes in visit volume and triage-level distribution, but these descriptive data cannot separate the intervention from the concurrent epidemic trajectory. The before-and-after design remains susceptible to secular trends.

Efficient triage processes have been associated with shorter delays and better ED operational measures [12]. Hospitals frequently respond to crowding by temporarily increasing clinical and support staffing [1], but staffing expansion may be difficult during widespread surges. A triage-triggered alarm is operationally distinct because it is designed to direct attention toward a small, high-risk group at the point of arrival. This targeted approach is consistent with evidence associating structured pediatric triage processes with improved prioritization of urgent patients [13].

Our findings also differ from reports that many physiologic monitor alarms in EDs are not associated with observable changes in care [14]. The intervention evaluated here was not a continuously generated monitor alarm; it was a discrete triage-triggered notification linked to an immediate workflow. Alarm performance may depend on clear thresholds, a defined response pathway, and adaptation to the local ED environment [15]. These features may also reduce unnecessary alerts and alarm fatigue. Hospital admission and actual or planned PICU admission were more frequent after implementation; however, these outcomes alone cannot establish whether post-intervention patients had greater illness severity because admission decisions may also reflect case mix, capacity, and practice patterns. All seven PED deaths in the matched actual-or-planned PICU subgroup occurred among patients who experienced IHCA before PICU transfer could be completed. When the sensitivity analysis was restricted to completed PICU admissions, PED mortality was zero in both periods. Thus, the numerical mortality difference using the composite denominator was driven entirely by the inclusion of planned-admission/IHCA cases and should not be interpreted as evidence of a mortality benefit associated with the intervention. The unmatched and matched mortality analyses diverged because matching excluded two pre-intervention deaths and no post-intervention deaths, reducing the comparison from seven versus two deaths to five versus two deaths. Unmatched visits had somewhat lower hospitalization and actual-or-planned PICU admission rates than matched visits in both periods, suggesting lower overall acuity based on these utilization measures. However, detailed physiologic and diagnostic data were unavailable to determine whether the unmatched visits differed systematically in clinical severity. The wide exact confidence interval in the matched analysis includes null value and reflects substantial imprecision; the mortality finding should therefore remain exploratory.

### Strengths and Limitations

This study has several important strengths. To our knowledge, this is the first study to evaluate a targeted intervention specifically designed to expedite care for triage level 1 patients during COVID-19 pandemic-related pediatric emergency department crowding. The pragmatic, low-complexity alarm system could be readily integrated into routine PED workflows. The large real-world cohort and propensity score matching, including adjustment for the availability of additional physician staffing support, improved comparability between the study periods. Furthermore, the study assessed clinical outcomes among critically ill patients and included triage level 2 waiting time as a balancing measure to evaluate potential unintended delays among other urgent patients.

Several limitations should be considered. First, the single-center, retrospective before-and-after design limits generalizability, does not permit causal inference, and cannot exclude secular changes. The pre- and post-intervention periods occurred during different phases of the Omicron wave and different calendar months; changes in visit volume, viral epidemiology, disease mix, acuity, or staffing pressure may therefore have confounded the association. Monthly visit and triage data were summarized descriptively, but no formal interrupted-time-series analysis was performed, and the independent effects of calendar time and the intervention could not be determined. Second, the complete-case analysis excluded 255 patients who left without being seen. Although the exclusion proportions were similar in the two study periods, detailed characteristics of excluded visits were unavailable, the missingness mechanism could not be assumed to be random, and selection bias remains possible. Third, the propensity model included only age group, sex, and physician staffing support. It did not balance triage level, and residual differences in triage mix may confound the observed waiting-time association; calendar month, arrival period, diagnosis, and repeated visits by the same child were also not modeled. Fourth, although the revised waiting-time bootstrap and permutation analyses retained matched pairs, triage-specific analyses compared marginal subgroups because paired visits often had different triage levels. The exploratory mortality analysis among the selected PICU subgroup did not fully account for matched-pair dependence. Fifth, the documentation timestamp may not correspond exactly to the start of bedside assessment, and automatic deactivation of the alarm on chart opening could have encouraged earlier electronic activity independent of clinical assessment. No independent bedside timestamp was available for validation; therefore, the observed reduction may reflect earlier electronic documentation, in whole or in part, rather than an earlier bedside assessment. Sixth, the composite of actual or planned PICU admission introduced outcome-dependent selection because all PED deaths occurred among patients with IHCA before transfer; the completed-PICU sensitivity analysis consequently contained no mortality events and could not provide an estimate for a comparative effect. Finally, the small number of deaths limited precision; the study did not assess post-PICU mortality, verify alarm activation for every eligible visit, calculate activation-to-chart-access response time, or evaluate long-term sustainability.

## 5. Conclusions

A triage-based emergency alarm was associated with a shorter median time to documented initial physician evaluation for triage level 1 pediatric visits. No statistically significant difference in triage level 2 waiting time was detected using matched-pair analysis. These observational findings support further evaluation with independent bedside timestamps, adjustment for triage mix and temporal trends, and patient-centered outcomes.

## Figures and Tables

**Table 1:** Characteristics of pediatric emergency department visits before and after implementation of the triage-based emergency alarm.

Characteristic	Pre-Intervention (*n* = 10,814)	Post-Intervention (*n* = 8810)	SMD	Pre-Intervention (*n* = 7467)	Post-Intervention (*n* = 7467)	SMD
Male sex, n (%)	5978 (55.3)	4759 (54.0)	−0.025	4168 (55.8)	4168 (55.8)	0.000
Age, years, mean ± SD	5.37 ± 4.41	4.11 ± 4.16	−0.295	4.0 ± 3.87	3.95 ± 4.03	−0.013
Age group, n (%)			—			—
<5 years	5686 (52.5)	6166 (70.0)	0.363	5402 (72.3)	5402 (72.3)	0.000
5–17 years	5128 (47.5)	2644 (30.0)	−0.363	2065 (27.7)	2065 (27.7)	0.000
Additional physician staffing support, n (%)	2153 (19.9)	1236 (14.0)	−0.157	1152 (15.4)	1152 (15.4)	0.000
Arrival time, n (%)			—			—
15:00–23:59	4960 (45.9)	3964 (45.0)	−0.018	3332 (44.6)	3557 (47.6)	0.060
00:00–14:59	5854 (54.1)	4846 (55.0)	0.018	4135 (55.4)	3910 (52.4)	−0.060
Triage level, n (%)						
Level 1	1183 (10.9)	691 (7.8)	−0.106	883 (11.8)	605 (8.1)	−0.125
Level 2	2922 (27.0)	2646 (30.0)	0.067	2496 (33.4)	2318 (31.0)	−0.051
Level 3	5624 (52.0)	4946 (56.1)	0.083	3413 (45.7)	4142 (55.5)	0.196
Level 4	965 (8.9)	492 (5.6)	−0.129	583 (7.8)	370 (5.0)	−0.117
Level 5	120 (1.1)	35 (0.4)	−0.082	92 (1.2)	32 (0.4)	−0.089

Note: SMD, standardized mean difference (post minus pre). Propensity score matching used age group, sex, and additional physician staffing support. Triage level was not included in the matching model.

**Table 2:** Waiting times and clinical outcomes before and after implementation of the triage-based emergency alarm.

Outcome	Pre-Intervention (*n* = 10,814)	Post-Intervention (*n* = 8810)	*p*-Value	Pre-Intervention (*n* = 7467)	Post-Intervention (*n* = 7467)	*p*-Value
Triage level 1 waiting time, min	15.0 ± 17.2	10.2 ± 8.9	<0.001	11.0 (6.8–17.2)	8.2 (5.7–12.5)	<0.001
Median difference, min (95% CI)	—	—	—	—	−2.82 (−3.48, −1.80)	—
Triage level 2 waiting time, min	18.0 ± 19.6	17.1 ± 19.2	0.089	12.4 (7.3–21.5)	11.8 (7.2–19.9)	0.088
Median difference, min (95% CI)	—	—	—	—	−0.58 (−1.37, 0.15)	—
Hospitalization, n (%)	1346 (12.5)	1414 (16.1)	<0.001	964 (12.9)	1210 (16.2)	<0.001
PICU admission or planned admission, n (%)	115 (1.1)	153 (1.7)	<0.001	87 (1.2)	139 (1.9)	<0.001
Completed PICU admission, n (%)	-	-	-	82 (1.1)	137 (1.8)	-
Planned PICU admission due to IHCA, n	-	-	-	5	2	-
PED mortality among completed PICU admissions, n/N (%)	-	-	-	0/82 (0)	0/137 (0)	NE
PED mortality among PICU admission or planned admission, n/N (%)	7/115 (6.1)	2/153 (1.3)	0.040	5/87 (5.7)	2/139 (1.4)	0.110

Note: Before matching, waiting times are mean ± SD; after matching, median (IQR). Matched waiting-time *p*-values use within-pair permutation tests; 95% CIs for differences between group medians use pair bootstrap resampling. Matched admission *p*-values use exact McNemar tests. The exploratory mortality comparison uses Fisher’s exact test.

## Data Availability

The data that support the findings of this study are available from the corresponding author upon reasonable request, subject to institutional and ethical restrictions.

## Supplementary Materials

The following supporting information can be downloaded at: https://www.mdpi.com/article/10.3390/children13101331/s1, Table S1: Monthly Pediatric Emergency Department Visit Volume and Triage-Level Distribution, April–October 2022.
