# Supplementary material for: Association of a Triage-Based Emergency Alarm System with Waiting Times and Clinical Outcomes in a Pediatric Emergency Department
Source: Children (Basel). 2026 Sep 30;13(10):1331. doi: 10.3390/children13101331 (PMC13702965; doi:10.3390/children13101331)
Supplement: Supplementary file 1 [file children-13-01331-s001.zip › children-4552220-supplementary.pdf]

Supplementary Table S1. Monthly Pediatric Emergency Department Visit Volume and Triage-Level Distribution, April-October 2022

Month	Total PED visits, n	Triage level 1, n (%)	Triage level 2, n (%)	Triage level 3, n (%)	Triage level 4, n (%)	Triage level 5, n (%)
April 2022	1843	138 (7.5)	403 (21.9)	1047 (56.8)	183 (9.9)	72 (3.9)
May 2022	4573	579 (12.7)	1121 (24.5)	2339 (51.1)	494 (10.8)	40 (0.9)
June 2022	2524	291 (11.5)	740 (29.3)	1290 (51.1)	193 (7.6)	10 (0.4)
July 2022	2013	184 (9.1)	684 (34.0)	1028 (51.1)	113 (5.6)	4 (0.2)
**Pre-intervention (Apr-Jul)**	**10,953**	**1192 (10.9)**	**2948 (26.9)**	**5704 (52.1)**	**983 (9.0)**	**126 (1.2)**
August 2022	2206	218 (9.9)	701 (31.8)	1180 (53.5)	96 (4.4)	11 (0.5)
September 2022	2900	185 (6.4)	928 (32.0)	1630 (56.2)	143 (4.9)	14 (0.5)
October 2022	3820	291 (7.6)	1042 (27.3)	2218 (58.1)	258 (6.8)	11 (0.3)
**Post-intervention (Aug-Oct)**	**8926**	**694 (7.8)**	**2671 (29.9)**	**5028 (56.3)**	**497 (5.6)**	**36 (0.4)**
**Overall (Apr-Oct)**	**19,879**	**1886 (9.5)**	**5619 (28.3)**	**10,732 (54.0)**	**1480 (7.4)**	**162 (0.8)**
